# Changes in Metabolic Profiles of Yellowtail (*Seriola quinqueradiata*) Muscle during Cold Storage as a Freshness Evaluation Tool Based on GC-MS Metabolomics

**DOI:** 10.3390/foods8100511

**Published:** 2019-10-18

**Authors:** Ryota Mabuchi, Miwako Adachi, Ayaka Ishimaru, Huiqing Zhao, Haruka Kikutani, Shota Tanimoto

**Affiliations:** 1 Faculty of Human Culture and Science, Prefectural University of Hiroshima, 1-1-71, Ujina-Higashi, Minami-ku, Hiroshima 734-8558, Japan; eh.mew.cf3@ezweb.ne.jp (M.A.); xoxo.ayaka.523.xoxo@gmail.com (A.I.); s-tanimoto@pu-hiroshima.ac.jp (S.T.); 2 Graduate School of Comprehensive Scientific Research, Prefectural University of Hiroshima, 1-1-71, Ujina-Higashi, Minami-ku, Hiroshima 734-8558, Japan; hermionek1220@gmail.com (H.Z.); sethm228@gmail.com (H.K.)

**Keywords:** metabolomics, GC-MS, muscles, cold storage, orthogonal partial least squares, *K* value

## Abstract

We applied metabolomics to the evaluation of yellowtail muscle as a new freshness evaluation method for fish meat. Metabolites from yellowtail ordinary and dark muscle (DM) stored at 0 °C and 5 °C were subjected to metabolomics for primary metabolites based on gas chromatography-mass spectrometry (GC-MS). For the annotated metabolites, we created statistically significant models for storage time prediction for all storage conditions by orthogonal partial least squares analysis, using storage time as the *y*-variable. DM is difficult to evaluate using the *K* value method, the predominant existing freshness evaluation method. However, in the proposed method, the metabolic component profiles of DM changed depending on storage time. Important metabolites determined from variables important for prediction (VIP) values included various metabolites, such as amino acids and sugars, in addition to nucleic-acid-related substances, especially inosine and hypoxanthine. Therefore, metabolomics, which comprehensively analyses different molecular species, has potential as a new freshness evaluation method that can objectively evaluate conditions of stored fish meat.

## 1. Introduction

Fish meat is generally recognized as a healthy food that is rich in high-quality proteins and *n*-3 fatty acids such as eicosapentaenoic acid and docosahexaenoic acid, and the demand for marine products is increasing worldwide. The freshness of fish meat deteriorates markedly with time, especially for raw fish. Therefore, accurate assessment methods of fish meat freshness are required. The existing freshness evaluation methods include physical measurements such as stiffness index, microbiological methods such as general viable count, sensory evaluations judged by human senses, and chemical methods such as volatile basic nitrogen and *K* value tests [1]. In particular, the *K* value, which is calculated according to the ratio of decomposition products of adenosine triphosphate (ATP), is a common chemical measurement method for fish meat [2]. However, the *K* value method has issues evaluating some fish species and tissues.

Metabolomics, the comprehensive analysis of metabolites, has been applied to the quality evaluation of various foods [3]. Metabolomic studies on fish include reports on the identification of biomarkers of fish disease [4], and the analysis of physiological responses due to environmental stress [5]. With regard to marine products, a previous report clarified the relationship between metabolites and microorganisms in the storage of mussels using nuclear magnetic resonance (NMR) metabolomics [6]. In another study, the authors used gas chromatography-mass spectrometry (GC-MS) metabolomics to differentially analyze four types of yellowtail muscle, and identified metabolites that contribute to muscle type differences [7]. In addition, according to the same method, metabolites related to taste attributes were clarified by a correlation analysis between the metabolites of ordinary muscle (OM) of four types of whitefish, and each taste value measured by an electronic tongue [8]. Thus, metabolomics can be effective for assessing the quality of aquatic products. However, to the best of the authors’ knowledge, metabolomics has not yet been applied to assessing the freshness of fish meat. Therefore, evaluating changes in metabolites due to fish meat storage using GC-MS-based metabolomics has great potential as a new freshness evaluation method.

In this study, we aimed to examine the effectiveness of metabolomics as a new method for evaluating the freshness of fish meat. Specifically, we analyzed the effects of the low-temperature storage of yellowtail dark muscle (DM) and OM on metabolic profiling. Furthermore, we compared the changes in *K* values and metabolic profiles caused by storage. The storage days model was created using an orthogonal partial least squares (OPLS) analysis, which uses storage days as the *y*-variable. This model is promising for this new freshness evaluation method. Interestingly, changes in the metabolic profile of DM depending on storage days were observed, which are difficult to evaluate with the *K* value method.

## 2. Materials and Methods

### 2.1. Chemicals

All reagents were special-grade chemicals. Methanol, chloroform, pyridine, and ribitol were purchased from Wako (Osaka, Japan). The derivatization reagents methoxyamine hydrochloride and *N*-methyl-*N*-(trimethylsilyl) trifluoroacetamide (MSTFA) were purchased from Sigma-Aldrich (St. Louis, MO, USA) and GL Sciences (Tokyo, Japan), respectively. The employed standards for nucleic-acid-related substances were hypoxanthine (Hx, Kanto Chemical, Tokyo, Japan), inosine (HxR, Nacalai Tesque, Kyoto, Japan), adenosine monophosphate (AMP, Tokyo Chemical Industry, Tokyo, Japan), inosine monophosphate (IMP) disodium hydrate (Tokyo Chemical, Tokyo, Japan), adenosine diphosphate (ADP) disodium salt (Oriental Yeast, Tokyo, Japan), and ATP disodium salt (Oriental Yeast, Tokyo, Japan).

### 2.2. Samples

Among the samples used in our previous report [9], the DM and OM of the dorsal part of the fish were used. Two yellowtail fish were purchased on three dates, i.e., July 14, September 29, and 5 November 2014, for a total of six fish (mean weight, 5.4 ± 1.2 kg). All six fish were reared by aquaculture and purchased at a local market in Hiroshima, Japan, where they were killed using the ikejime fish-slaughtering method. They were then transported on ice to the laboratory within 8 h. Muscle samples of the same type from the two fish purchased on the same date were minced together using a food processor (MK-K60P, Panasonic, Kadoma, Japan). The minced muscle samples were kept in ice storage (0 °C) for 14 days or at 5 °C for 7 days. Samples were then stored at −80 °C until analysis. As previously reported, samples were stored under conditions in which the number of viable bacteria did not increase significantly [9].

### 2.3. Quantitative Analysis of Nucleic-Acid-Related Substances and Calculation of K Values Using Ultra-Performance Liquid Chromatography (UPLC)

The extraction of nucleic-acid-related substances was performed according to a partially modified version of the method reported by Murata and Sakaguchi [10]. In short, 4 mL of 10% perchloric acid was added to 2.0 g of each fish meat sample; the mixture was homogenized on ice (T 25 digital ULTRA-TURRAX, IKA, Deutschland, Germany) (5000 rpm, 2 min) and centrifuged (10,000 rpm, 5 min, 3 °C). The supernatant after centrifugation was transferred to another container. Subsequently, 4 mL of 5% perchloric acid was added to the precipitate; the mixture was homogenized again on ice (5000 rpm, 2 min) and centrifuged (10,000 rpm, 5 min, 3 °C), and the obtained supernatant was mixed with the previous supernatant. After neutralization with potassium hydroxide and centrifugation (10,000 rpm, 5 min, 3 °C), the supernatant was removed, made up to 25 mL with distilled water, and stored at −80 °C until analysis. After analysis, the sample was diluted 10-fold, filtered (pore size: 0.45 μL), and subjected to UPLC analysis.

The extracted nucleic-acid-related substances were analyzed by a UPLC system with a tunable ultraviolet detector (ACQITYTM Ultra Performance LC, Waters, Tokyo, Japan). The employed UPLC column was a Kinetex Evo C18 (1.7 μm, 50 mm × 2.1 mm − 100 A, Shimadzu LC, Kyoto, Janpa). The temperature of the column was set to 40 °C, and the mobile phase by filtration was 100 mmol/L phosphoric acid and 150 mmol/L triethylamine/distilled water = 100/1 (*v*/*v*). The flow rate was 0.9 mL/min, injection volume was 1 μL, and measurement wavelength was 260 nm. Identification of detected nucleic-acid-related substances was performed by comparison with the retention time of each standard substance, and quantification was performed by comparison with the peak area of each standard substance.

The *K* values were calculated by the following equation based on quantitative values obtained from the analysis of nucleic-acid-related substances by UPLC: *K* (%) = (HxR + Hx)/(ATP + ADP + AMP + IMP + HxR + Hx) × 100 [11].

### 2.4. GC-MS and Multivariable Analysis

Samples for GC-MS analysis were 1 g of uniform minced fish freeze-dried overnight in a lyophilizer. The dried samples were milled to a powder at 25,000 rpm for 30 s using a mill (TUBE-MILLC S001, IKA, Deutschland, Germany).

GC-MS analysis was performed according to previous reports [7,8]. To each 50 mg of powdered sample, mixed solutions of methanol/ultrapure water/chloroform (2.5/1/1 *v*/*v*/*v*, 1 mL) and ribitol (internal standard, 0.2 mg/mL, 60 µL) were added. After stirring for 5 min, the mixture was centrifuged (16,000× *g*, 0 °C, 5 min). Ultrapure water (400 µL) was added to 800 µL of the supernatant, followed by stirring for 1 min and then centrifugation (16,000× *g*, 0 °C, 5 min). A 400 µL portion of the supernatant was concentrated for 1 h using a centrifugal evaporator (CVE-2000, Eyela, Tokyo, Japan) and then freeze-dried overnight. Methoxyamine hydrochloride solubilized with pyridine (20 mg/mL, 50 µL) was added to the freeze-dried sample, and oxime formation was carried out by reacting at 30 °C for 90 min. MSTFA (100 µL) was further added, and trimethylsilylation was carried out by reacting at 37 °C for 30 min. The derivatized samples were analyzed by GC-MS using a GCMS-QP2010 Ultra system (Shimadzu, Kyoto, Japan) equipped with an Agilent J&W DB-5 column (length 30 m, internal diameter 0.25 mm, film thickness 1.00 µm, Agilent Technologies, Santa Clara, CA, USA). The GC oven temperature began at 100 °C, remained for 4 min, increased to 320 °C at 10 °C/min, and remained for 11 min, and the injection port temperature was 280 °C. The derivatized sample (1 µL) was injected in split injection mode with a split ratio of 10:1. Helium was employed as the carrier gas at a constant linear velocity of 39.0 cm/s, and the purge flow rate was 5 mL/min. Quadrupoles were used for MS mass separation, and electron impact was used for ionization. The ion source temperature was 200 °C, interface temperature was 280 °C, and ionization voltage was 70 eV. Measurements were carried out in scan mode in the range of 45–600 m/z. Retention time correction of peaks (retention index) was carried out based on the retention time of a standard alkane series mixture (C-6 to C-33) using the automatic adjustment of retention time function of the Shimadzu GCMSsolution software. Annotation of peaks was performed using the commercially available GC/MS Metabolite Component Database Ver. 2 (Shimadzu, Kyoto, Japan), which contains a mass spectral library. Peaks were annotated under the condition of possessing a similarity index of more than 80% and a target ion with a confirmation ion ratio of ≥50% in absolute tolerance, while peaks with a similarity index of <80% were regarded as belonging to unknown metabolites.

SIMCA 14 (MKS Instruments, Andover, MA, USA) was used for multivariate analysis. The data sets consisted of sample name in column 1, *y* variables (storage days) in column 2, and each corrected peak intensity of the annotated components in subsequent columns. Data pre-processing was performed using unit-variance scaling (UV) and Pareto scaling (Par). Principal component analysis (PCA) was used to acquire an overview of the data. OPLS was used to analyze the influence of storage time for each storage temperature on metabolic component profiles and to create a prediction model of storage time. Evaluation of the model obtained by OPLS analysis was considered statistically significant at *R*^2^*Y* ≥ 0.65 and *Q*^2^*Y* ≥ 0.5 [12,13]. Variables important for prediction (VIP) values were calculated to identify the characteristic metabolites that changed with storage. Metabolites with a VIP value of 1.0 or higher were ranked as metabolites with a high VIP [14]. Coefficients were calculated to confirm the correlativity of each metabolite.

## 3. Results

### 3.1. K Values

**Figure 1:**
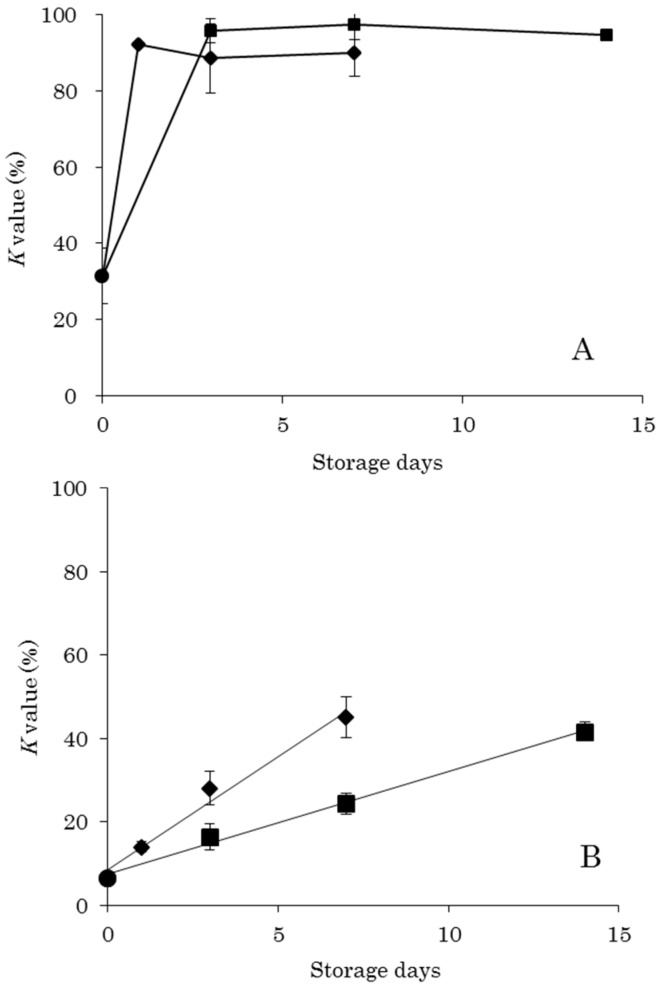
Changes in *K* value by storage of yellowtail muscle. (**A**) Dark muscle (DM), ● no storage (control), ■ 0 °C storage, ◆ 5 °C storage; and (**B**) Ordinary muscle (OM), ● no storage (control), ■ 0 °C storage (regression line: *y* = 2.45*x* + 7.50, *R^2^* = 0.99), ◆ 5 °C storage (regression line: *y* = 5.45*x* + 8.40, *R^2^* = 0.98).

*K* values were calculated from each quantitative value obtained from the UPLC analysis of nucleic-acid-related substances. The scatter plot in Figure 1 shows the relationship between *K* value and storage days. DM had a *K* value of 31.3% in the control sample, and that of DM stored at 0 °C exceeded 90% in 3 days. Subsequent storage for 7 days and 14 days did not result in an increase in *K* value (Figure 1A). Storage of DM at 5 °C also resulted in a *K* value exceeding 90% in one day, and no increase was observed after storage for 3 days and 7 days. The OM *K* value was 6.4% in the control samples and increased depending on storage time both at 0 °C and at 5 °C (Figure 1B). Regression equations were determined as *y* = 5.45*x* + 8.34 (*R^2^* = 0.98) at 0 °C and *y* = 2.45*x* + 7.50 (*R^2^* = 0.99) at 5 °C, both of which showed linearity. The above-mentioned change in *K* value caused by low-temperature storage of DM and OM is similar to previously reported results [10].

### 3.2. GC-MS Analysis

As a result of exhaustive detection, 120 metabolites were annotated under the conditions of this study. A list of metabolites annotated in each sample is shown in Table S1. The number of annotated metabolites in DM was 52 for the control, 78 for storage at 0 °C, and 82 for storage at 5 °C; for OM, the number was 56 for the control, 83 for storage at 0 °C, and 92 for storage at 5 °C. The number of metabolites tended to increase upon going from storage at 0 °C to storage at 5 °C. Additionally, 128 peaks of unknown metabolites were detected, 99 for DM and 99 for OM (Table S2).

### 3.3. PCA

**Figure 2:**
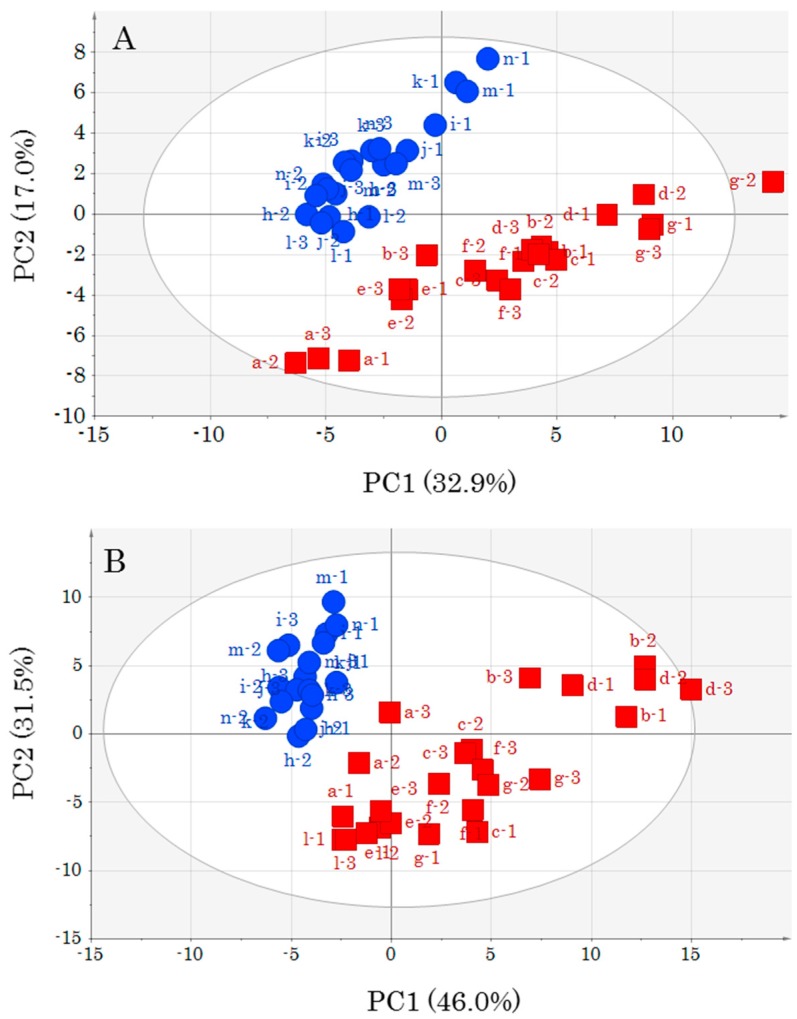
Score plots obtained by principal components analysis (PCA) of all samples using the data set in Table S1. (**A**) Score plots obtained using unit variance-scaling (UV); and (**B**) score plots obtained using Pareto-scaling (Par) ●OM, ■DM. Lower-case letters and numbers indicate sample ID (Table S1).

Multivariate analysis (PCA) with SIMCA was performed using the data sets generated from the annotated metabolites (Table S1). First, PCA was performed using data from all samples to obtain an overall image. The score plot obtained by PCA is shown in Figure 2. The PCA by UV was 32.9% for the first principal component (PC1) and 17.0% for the second principal component (PC2), and the cumulative contribution percentage was 49.8%. The PCA by Par was 46.0% for PC1 and 31.5% for PC2, and the cumulative contribution percentage was 77.5%. As shown in Figure 2A, OM was grouped around negative PC1 and positive PC2, and DM was grouped around positive PC1 and negative PC2, indicating differences due to muscle type. This result agrees with that of the previous report [7] on site discrimination of yellowtail muscle. Furthermore, it was possible to discriminate the site regardless of storage or lack thereof.

The OM control was located in negative PC1 and the center of PC2, from which samples after storage shifted toward positive for both PC1 and PC2. Thus, storage effects tended to be reflected by the metabolic component profile. As for DM, the control was located in negative PC1 and PC2, and samples after storage shifted toward positive PC1. The change in the metabolic component profile due to storage was larger for DM than for OM. This tendency was also present in the PCA results obtained by Par (Figure 2B). The loading plot obtained by PCA is shown in Figure S1. In addition, unknown metabolite data (Table S2) were added to those of annotated metabolites (Table S1), and PCA was performed using all peaks (Figure S2). The contribution percentage was close to that obtained when PCA was performed with annotated metabolites only. In addition, the obtained loading plot shows that most metabolites correlated with DM and OM were annotated metabolites, which was particularly noticeable for PCA using Par.

**Figure 3:**
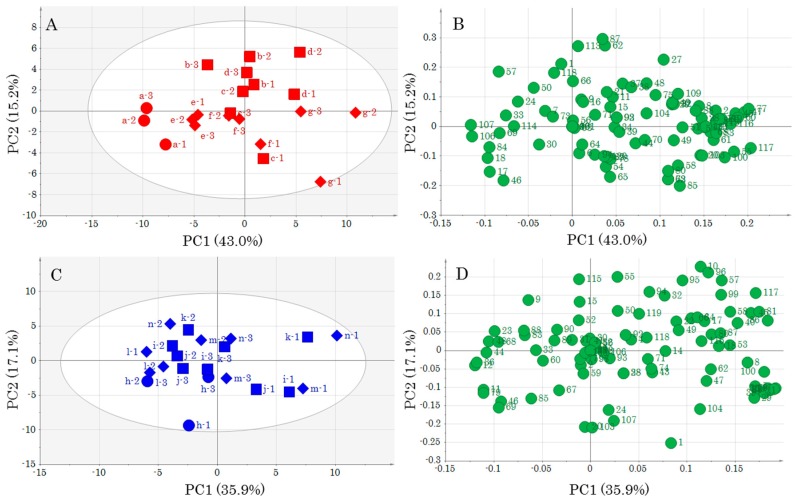
Score plots and loading plots obtained by PCA of each muscle part using UV scaling. (**A**) Score plot for DM. ● No storage (control), ■ 0 °C storage, ◆ 5 °C storage; (**B**) loading plot for DM; (**C**) score plot for OM. ● No storage (control), ■ 0 °C storage, ◆ 5 °C storage; and (**D**) loading plot for OM. Lower-case letters and numbers in (**A**,**C**) indicate sample ID, and numbers in (**B**,**D**) indicate number of metabolites (Table S1).

Next, PCA was performed for each muscle type (Figure 3 and Figure S3) using the data set in Table S1. The score plot obtained by PCA of DM using UV showed that the control was positioned in negative PC1, which shifted toward the positive direction with each day of storage. Changes in metabolic component profiles with storage days were observed. Storage at 0 °C tended to be located in positive PC2 and at 5 °C in negative PC2. The control samples were located in negative PC1 and reflected storage effects toward the positive direction. These results indicate that differences in storage time and temperature alter the metabolic component profiles of DM. The PCA of OM using UV also showed changes in metabolic component profiles similar to that of DM (Figure 3C). The loading plots in Figure 3B,D had more metabolites located in positive PC1 than in negative. Furthermore, some metabolites were increased by storage. In particular, in the loading plot obtained by PCA using Par, taurine, inosine, etc., in DM, and histidine, creatinine, etc., in OM were shown to be characteristic metabolites. Phosphoric acid, lactic acid, etc., were characteristic metabolites common to DM and OM (Figure S3).

### 3.4. OPLS

Multivariate analysis (OPLS) with SIMCA was performed using the data sets generated from annotated metabolites (Table S1). Table 1 shows the results of OPLS analysis performed for each storage temperature on DM and OM. The score plots obtained by OPLS were separated according to storage time under all conditions. This separation changed from negative *t* [1] for the control samples toward the positive direction depending on storage time (Figure S4). In general, an *R*^2^*Y* value of 0.65 or more and *Q*^2^*Y* of 0.5 or more indicate a satisfactory ability for quantitative prediction, and thus all prediction models met the evaluation criteria. Therefore, this model can predict storage time (Table 1, Figure S5). A regression equation in which the relationship between actual values (vertical axis), and predicted values (horizontal axis) of this storage time prediction model was plotted and showed good *R*^2^ values for all models. In particular, OM with Par at 5 °C storage had the highest *R*^2^.

VIP values were calculated in order to identify important metabolites contributing to each model (Table S3). In addition, coefficients were calculated to confirm the correlation of each metabolite with a high VIP value (Table S4). The metabolites with VIP values of 1.0 or more were regarded as important (high VIP value). Many metabolites showed high VIP values in each model, showing that cold storage alters a variety of metabolites. The 10 highest VIP values were extracted and are listed in Table 2. Metabolites with high VIP values were different between DM and OM. This was influenced not only by differences in the originally existing metabolites, but also changes in the resulting metabolites due to storage. Differences in scaling and storage temperature also affected changes in metabolites.

## 4. Discussion

The purpose of this study was to analyze changes in metabolic component profiles induced by low-temperature storage using GC-MS and to examine its potential for use as a new freshness evaluation method.

The *K* value method, which is typically used for chemical freshness evaluation, has difficulties in evaluating DM. By *K* value standards, raw food (sashimi) is acceptable to eat at a value of 20% or less, heat-treated food is acceptable in the range of 20% to 50%, and a value of 60% or more is an indicator of food spoilage [2]. The *K* values in this study were less than 50% after 14 days of storage at 0 °C and 7 days at 5 °C in OM (Figure 1). Therefore, the storage conditions implemented in this study were within the acceptable range for heated food. Furthermore, storage for 3 days at 0 °C and 1 days at 5 °C resulted in *K* values of less than 20%, indicating that these storage conditions allowed for raw consumption. On the other hand, the DM *K* values were over 20%, even before storage, and storage at 0 °C for 3 days and at 5 °C for 1 days resulted in values of 90% or more. These storage conditions were thus evaluated by the *K* value method as yielding rotten food. In a previous report, viable cell count was determined by a colony-forming units assay on the same samples as used in this study [9]. No significant increase in viable cell count was observed in DM under the same storage conditions. Therefore, as evaluated by viable cell count data, DM was acceptable to eat under these conditions. Thus, the results of this study also reveal that it is difficult to accurately evaluate the freshness of DM using the *K* value as an indicator.

Metabolomics has been applied to the quality evaluation of various foods, but there have been very few reports on its application to the quality evaluation of fish meat. One study evaluated metabolic profiles of *Sparus aurata* under ice storage using ^1^H-NMR metabolomics [15]. However, no studies have evaluated changes in metabolic component profiles depending on storage times as in this study. We propose a prediction model for storage time created by OPLS analysis as a new freshness evaluation method. This study is the first to establish a freshness assessment method based on fish metabolomics.

PCA, which was conducted to understand the appearance of the data, revealed changes in metabolomics profiles due to muscle type differences and storage conditions. In particular, it is very interesting that a difference in storage temperature contributed to a difference in metabolic component profiles. PCA performed by adding unknown metabolite data to annotated metabolite data featured a contribution percentage close to that obtained when only annotated metabolites were employed, i.e., the presence of numerous unknown metabolites was not important for highlighting differences between DM and OM. However, as some of the unknown metabolites may be important for a deeper understanding of the yellowtail metabolism, their identification is an important task that should be addressed in the future. However, identification of all unknown metabolites by GC-MS analysis alone is difficult. Therefore, we are considering the implementation of complex metabolomics through the use of other metabolomics tools such as LC-MS and CE-MS.

An OPLS model was created for each storage temperature as it became clear that storage temperature affected the metabolic component profile. In addition, the data pre-treatment for OPLS analysis has yielded different important metabolites depending on the choice of scaling method [8]. Therefore, in this study, we created prediction models using two different scaling methods, UV and Par. Statistically meaningful prediction models were created for all conditions. As the prediction models use storage days as *y*-variables, they can predict how many days a sample has been stored. However, the conditions that can be predicted are limited. That is, only storage at 0 °C up to 14 days and at 5 °C up to 7 days can be predicted. Prediction models for other temperature zones and long-term storage are topics for further study. However, considering the actual consumption of fish meat, it is not realistic for it to be stored for long periods above 5 °C. Therefore, the storage conditions in this study may be sufficient for freshness assessment. Yellowtail was used in this study, and thus application to other fish species is also a future research subject. Nonetheless, we determined changes in the metabolic component profiles of cold-stored yellowtail OM and DM depending on storage time and were able to create subsequent prediction models. In this regard, it can be said that metabolomics is an effective new freshness evaluation method. Especially, DM, which is difficult to evaluate using the *K* value method, was successfully modeled for the prediction of storage time using metabolomics. Therefore, metabolomics allows for objective evaluation of the freshness of DM.

The creation of a prediction model for storage time can also identify important metabolites that change with storage. Therefore, it is possible to metabolically consider the relationship between different fish meat metabolic components due to storage.

A large amount of ATP is present in fish muscle. After death, ATP diminishes and eventually disappears. Therefore, a decrease in ATP is an indicator of a decline in the freshness of fish meat. The degradation of ATP that accompanies the decrease in freshness follows the order of ATP→ADP→AMP→IMP→HxR→Hx. In metabolomics by GC-MS, IMP, HxR, and Hx were annotated in this study (Table S1). Although IMP was not detected in many samples, HxR and Hx showed high VIP values in the prediction model by most OPLS analyses. Previously, Murata and Sakaguchi [10] stored yellowtail muscle in ice storage and evaluated the nucleic-acid-related substances and *K* values. The results showed that HxR and Hx increased in OM with storage time. In DM, HxR increased in the first 2 days of storage but did not change thereafter. On the other hand, Hx increased over 12 days of storage. Similarly, in the present study, HxR and Hx showed a positive correlation with the number of storage days, which was consistent with the results of the previous study. However, in addition to these nucleic-acid-related substances, metabolites of various chemical species also showed high VIP values. Therefore, the results of this study also indicate that changes in metabolites due to storage of fish meat cannot be explained by nucleic-acid-related substances alone. In the OPLS analysis by Par of OM, phosphoric acid showed the highest VIP value at both 0 °C and 5 °C, which was considered to arise from phosphate generated by the degradation of ATP. Since phosphoric acid showed remarkably high VIP values, even compared with other important metabolites, it may be suitable as a marker for freshness evaluation by reflecting the effects of OM storage.

Many amino acids showed higher VIP values in DM compared with those in OM. In a previous study analyzing changes in free amino acids due to the storage of yellowtail muscle, in OM, there was no change due to storage (40 days) in most free amino acids, but in DM, increases in many free amino acids were observed [16]. Furthermore, an increase in alanine or branched-chain amino acids has been demonstrated as an indicator of proteolysis [17]. In particular, in the prediction model of DM using UV, alanine, valine, and isoleucine showed high VIP values. Therefore, the results of this study suggest that proteins were degraded during storage under the studied conditions.

Some monosaccharides such as glucose and galactose also showed high VIP values. In some studies, monosaccharides have been shown to increase by the storage of aquatic products. For example, an increase in glucose due to 5 °C storage of mussels and increase in ribose due to ice storage of flounder have been reported [6,18]. Therefore, monosaccharides can also be considered as reflecting the effects of aquatic product storage.

As described above, storage of fish meat causes changes in various metabolic components, and the changing components differ depending on the muscle type and storage temperature. Therefore, metabolomics, which can comprehensively analyze different molecular species, has potential as a new freshness evaluation method that can objectively evaluate the conditions of fish meat after storage. In this study, we examined storage at 0 °C and 5 °C. Since the metabolic profiles at 0 °C and 5 °C are different, we can readily assume that the metabolic components will further differ at lower or higher temperatures. The predictive model in this study can only evaluate the DM and OM in yellowtail at 0 °C and 5 °C, which is a limitation; hence, for future applications there is a need to conduct larger-scale storage experiments over a wider temperature range and with a variety of fish species.

## 5. Conclusions

The purpose of this study was to examine the feasibility of a method for evaluating the freshness of fish meat using metabolomics based on GC-MS. We used low-temperature storage samples of yellowtail OM and DM as models. As a result of OPLS analysis, which analyzed storage days as the *y*-variable, significant changes in the metabolic profiles depending on storage days were recognized under all conditions. Interestingly, the freshness of DM could be evaluated, which is difficult to evaluate using the *K* value, an existing chemical freshness evaluation method. This is the first study to apply GC-MS-based metabolomics to the assessment of fish meat freshness.

## Figures and Tables

**Table 1:** Evaluation of models obtained by orthogonal partial least squares (OPLS).

Sample		Scaling	*A* ^a^	*N* ^b^	*R^2^X*	*R^2^Y*	*Q^2^Y*	Regression Line			
								*y*	*R^2^*	*RMSEE*	*RMSEcv*
Dark muscle	0 °C	UV	1+0+0	12	0.442	0.742	0.668	0.983*x* + 0.683	0.755	2.920	3.101
		Par	1+1+0	12	0.806	0.727	0.535	0.950*x* + 0.551	0.732	3.164	3.683
	5 °C	UV	1+1+0	12	0.616	0.952	0.865	1.008*x* + 0.114	0.954	0.680	1.111
		Par	1+4+0	12	0.941	0.959	0.909	0.996*x* − 0.036	0.960	0.765	0.949
Ordinary muscle	0 °C	UV	1+1+0	12	0.521	0.874	0.611	0.997*x* + 0.707	0.891	2.151	3.471
		Par	1+2+0	12	0.722	0.898	0.701	0.999*x* + 0.016	0.898	2.052	2.865
	5 °C	UV	1+1+0	12	0.578	0.884	0.778	0.973*x* + 0.375	0.898	1.054	1.455
		Par	1+5+0	12	0.981	0.995	0.955	1.002*x* − 0.050	0.995	0.294	0.659

^a^*A* = number of models. ^b^ *N* = number of samples used in producing models. UV = unit variance-scaling; Par = Pareto-scaling; *RMSEE* = root mean square errors of estimation; and *RMSEcv:* root mean square errors of cross-validation.

**Table 2:** Metabolites which showed high variables important for prediction (VIP) values obtained from OPLS analysis.

Muscle Type	Dark Muscle						Ordinary Muscle					
Storage Tem.	0 °C			5 °C			0 °C		5 °C			
Scaling	UV						UV					
VIP Rank	metabolite	VIP values	Coefficient	metabolite	VIP values	Coefficient	metabolite	VIP values	Coefficient	metabolite	VIP values	Coefficient
1	Methionine-2TMS	2.45	0.04	Glyceric acid-3TMS	1.94	0.04	Arabinose-meto-4TMS	2.70	0.08	Arabinose-meto-4TMS	2.15	0.05
2	Glycerol-3TMS	2.22	0.03	Glycerol-3TMS	1.93	0.03	Ribose-meto-4TMS	2.70	0.08	Ribose-meto-4TMS	2.06	0.04
3	Galactose-meto-5TMS(2)	2.13	0.03	Tyrosine-3TMS	1.92	0.03	Ribose-4TMS(4)	2.38	0.06	Uracil-2TMS	2.05	0.04
4	Phenylalanine-2TMS	2.03	0.03	Ornithine-4TMS	1.89	0.04	Inosine-4TMS	2.24	0.07	Inosine-4TMS	1.98	0.05
5	Valine-2TMS	2.02	0.03	Hypoxanthine-2TMS	1.85	0.04	Uracil-2TMS	2.18	0.07	Hypoxanthine-2TMS	1.93	0.07
6	Uracil-2TMS	2.01	0.03	Uracil-2TMS	1.82	0.02	Ribulose-meto-4TMS	2.04	0.05	Fructose 1-phosphate-meto-6TMS(1)	1.91	0.06
7	Threonine-3TMS	2.00	0.03	Serine-3TMS	1.78	0.03	Glyceric acid-3TMS	1.93	0.03	Fructose 1-phosphate-meto-6TMS(2)	1.91	0.06
8	Isoleucine-2TMS	1.97	0.04	Isoleucine-2TMS	1.77	0.03	Niacinamide-TMS	1.85	0.03	*N*-Acetylaspartic acid-2TMS	1.83	−0.05
9	Hypoxanthine-2TMS	1.97	0.03	Methionine-2TMS	1.75	0.03	Phenylalanine-2TMS	1.83	0.03	Glycerol 3-phosphate-4TMS	1.79	−0.05
10	2-Aminoethanol-2TMS	1.95	0.03	Valine-2TMS	1.74	0.04	Ascorbic acid-4TMS	1.76	-0.07	Ascorbic acid-4TMS	1.79	−0.05
Scaling	Par						Par					
VIP Rank	metabolite	VIP values	Coefficient	metabolite	VIP values	Coefficient	metabolite	VIP values	Coefficient	metabolite	VIP values	Coefficient
1	Galactose-meto-5TMS(1)	6.15	0.36	Galactose-meto-5TMS(1)	4.58	−0.01	Phosphoric acid-3TMS	5.42	0.19	Phosphoric acid-3TMS	6.57	0.25
2	Taurine-3TMS	4.70	−0.08	Taurine-3TMS	4.53	−0.11	Lactic acid-2TMS	5.12	−0.46	Lactic acid-2TMS	3.91	−0.46
3	Tagatose-5TMS(5)	3.41	0.02	Tagatose-5TMS(5)	4.47	0.20	Inosine-4TMS	4.34	0.95	Inosine-4TMS	3.42	0.58
4	Mannose-meto-5TMS(1)	3.16	0.05	Mannose-meto-5TMS(1)	3.03	−0.07	Ascorbic acid-4TMS	1.81	−0.36	Histidine-3TMS	2.60	0.25
5	Galactose-meto-5TMS(2)	2.95	0.26	Galactose-meto-5TMS(2)	2.70	0.26	Glucose-meto-5TMS(1)	1.77	0.03	Creatinine-3TMS	2.07	0.31
6	Phosphoric acid-3TMS	2.74	−0.05	Inosine-4TMS	2.13	-0.13	Mannose-meto-5TMS(2)	1.77	0.03	Galactose-meto-5TMS(1)	1.88	−0.01
7	Glucose-meto-5TMS(2)	2.16	0.11	Phosphoric acid-3TMS	1.68	0.10	Fucose-4TMS(2)	1.73	0.27	Glucose-meto-5TMS(1)	1.87	−0.01
8	Glucose-meto-5TMS(1)	1.72	0.10	Phenylalanine-2TMS	1.64	0.16	Creatinine-3TMS	1.72	−0.41	Mannose-meto-5TMS(2)	1.87	−0.01
9	Lactic acid-2TMS	1.64	−0.09	Glycerol-3TMS	1.63	0.20	Tagatose-5TMS(5)	1.68	−0.03	Mannose-meto-5TMS(1)	1.79	−0.06
10	Phenylalanine-2TMS	1.06	0.10	Hypoxanthine-2TMS	1.52	0.18	Galactose-meto-5TMS(1)	1.67	−0.01	Tagatose-5TMS(5)	1.79	−0.06

UV = unit variance-scaling; and Par = Pareto-scaling.

## Supplementary Materials

The following are available online at https://www.mdpi.com/2304-8158/8/10/511/s1, Table S1: List of metabolites detected by GC-MS analysis. Table S2: List of unknown metabolites detected by GC-MS. Table S3: List of VIP values obtained by OPLS analysis. Table S4: List of coefficients obtained by OPLS analysis. Figure S1: Loading plots obtained by PCA of all samples. Figure S2: Score plots and loading plots obtained by PCA of all samples using the data sets in Table S1 and S2. Figure S3: Score plots and loading plots obtained by PCA of each muscle part using Par scaling. Figure S4: Score plots and loading plots obtained by OPLS analyzed under each condition. Figure S5: Regression equations of prediction models obtained by OPLS.
